# Lactacystin-induced kidney fibrosis: Protection by melatonin and captopril

**DOI:** 10.3389/fphar.2022.978337

**Published:** 2022-09-13

**Authors:** Kristina Repova, Peter Stanko, Tomas Baka, Kristina Krajcirovicova, Silvia Aziriova, Jaroslav Hrenak, Andrej Barta, Stefan Zorad, Russel J. Reiter, Michaela Adamcova, Fedor Simko

**Affiliations:** ^1^ Institute of Pathophysiology, Faculty of Medicine, Comenius University, Bratislava, Slovakia; ^2^ Medbase, Bern, Switzerland; ^3^ Institute of Normal and Pathological Physiology, Centre of Experimental Medicine, Slovak Academy of Sciences, Bratislava, Slovakia; ^4^ Institute of Experimental Endocrinology, Biomedical Research Center, Slovak Academy of Sciences, Bratislava, Slovakia; ^5^ Department of Cell Systems and Anatomy, UT Health San Antonio, Long School of Medicine, San Antonio, TX, United States; ^6^ Department of Physiology, Faculty of Medicine in Hradec Kralove, Charles University, Hradec Kralove, Czechia; ^7^ 3rd Department of Internal Medicine, Faculty of Medicine, Comenius University, Bratislava, Slovakia

**Keywords:** lactacystin, melatonin, captopril, kidney injury, fibrotic remodeling, cardiorenal damage

## Abstract

Lactacystin is a specific proteasome inhibitor that blocks the hydrolysis of intracellular proteins by ubiquitin/proteasome system inhibition. The administration of lactacystin to rats induced hypertension and remodeling of the left ventricle and aorta. This study tested whether lactacystin induces structural and fibrotic rebuilding of the kidneys and whether melatonin and captopril can prevent these potential changes. Six weeks of lactacystin administration to rats increased their average systolic blood pressure (SBP). In the kidneys, lactacystin reduced glomerular density, increased the glomerular tuft area, and enhanced hydroxyproline concentrations. It also elevated the intraglomerular proportion including the amounts of collagen (Col) I and Col III. Lactacystin also raised the tubulointerstitial amounts of Col I and the sum of Col I and Col III with no effect on vascular/perivascular collagen. Six weeks of captopril treatment reduced SBP, while melatonin had no effect. Both melatonin and captopril increased glomerular density, reduced the glomerular tuft area, and lowered the hydroxyproline concentration in the kidneys. Both drugs reduced the proportion and total amounts of intraglomerular and tubulointerstitial Col I and Col III. We conclude that chronic lactacystin treatment stimulated structural and fibrotic remodeling of the kidneys, and melatonin and captopril partly prevented these alterations. Considering the effect of lactacystin on both the heart and kidneys, chronic treatment with this drug may be a prospective model of cardiorenal damage suitable for testing pharmacological drugs as protective agents.

## Introduction

Hypertension and diabetes are the leading causes of chronic kidney disease (CKD) (Hamrahian and Falkner, 2017). The pathogenesis of CKD in hypertension is multifactorial, involving genetic alterations, oxidative stress, endothelial dysfunction, and renin-angiotensin-aldosterone system (RAAS) disturbances (Mennuni et al., 2014). These factors promote inflammation, fibrosis, and both a reduced nephron count and a lower glomerular filtration rate (GFR) (Schlondorff, 2008). CKD is associated with a worsening cardiovascular prognosis and constitutes a serious health and social condition. Thus, there is a continuous search for new ways of protection in experimental models of hypertension.

Cardiovascular homeostasis is tightly bound to proper protein turnover, which is controlled by the ubiquitin-proteasome system (UPS). Proteasomes are multisubunit protease complexes that degrade damaged proteins in all parts of the cells (Pagan et al., 2013). The proteasomes specific to the heart involve a number of proteins with various biological impacts, such as atrogin (known as muscle atrophy F-box) participating in myocardial remodeling (Li et al., 2004); murine double minute 2 (MDM2), a ubiquitin ligase that mediates p53 participating on myocardial hypertrophy modulation (Chatterjee et al., 2011); the calcineurin-nuclear factor of the activated T cells (NFAT) pathway that mediates cardiac remodeling (Tang et al., 2010); sarcomere-associated protein MuRF1 associated with heart failure (Willis et al., 2009); or Nedd4 containing E3 ligase controlling the biological impact of multifunctional vascular endothelial growth factor (VEGF) (Murdaca et al., 2004). In the kidney, Nedd4L/Nedd4-2 seems to participate in distal nephron salt sensitivity (Ishigami et al., 2020), and ubiquitin-conjugating enzyme E2 contributes to HUWE1-mediated degradation of tubulointerstitial fibrosis (Wang et al., 2022). Pharmacological interference with proteasomes is emerging as a potential approach to influence various pathological processes.

Lactacystin is a proteasome inhibitor that blocks the hydrolysis and degradation of intracellular proteins by the UPS (Craiu et al., 1997). Chronic lactacystin administration induces a mild but significant rise in systolic blood pressure (BP) along with fibrotic remodeling of the left ventricle (LV) in Wistar rats (Simko et al., 2017), and hypertrophy of the aorta in L-NAME (L-NG-nitro arginine methyl ester)-induced hypertension (Vrankova et al., 2010). Several hypothetical mechanisms for hemodynamic alterations and heart and vessel damage by lactacystin have been proposed, such as enhanced oxidative stress (Vrankova et al., 2010; Huseby et al., 2016; Parajuli, 2019), decreased NO bioavailability (Simko et al., 2017; Sharma et al., 2020), sympathetic nervous system activation (Congo Carbajosa et al., 2015) and modification of various cytosolic, nuclear, and myofibrillar protein turnovers (Mearini et al., 2008). However, data on lactacystin’s renal effects are not available.

The aim of this study was to test whether lactacystin induces structural and fibrotic rebuilding of the kidneys. Furthermore, we sought to determine whether melatonin prevents these potential alterations. Melatonin (N-acetyl-5-methoxytryptamine), the main product of the pineal gland, not only regulates biological circadian rhythms (Reiter et al., 2020), but exerts various pleiotropic protective effects on the heart, vasculature (Reiter et al., 2010; Simko et al., 2019, 2020; Domínguez-Rodríguez et al., 2021; Tobeiha et al., 2022) and kidneys (Russcher et al., 2012; Hrenák et al., 2013; Hrenak et al., 2015; Shi et al., 2019). The effects of melatonin were compared with captopril, a classical angiotensin-converting enzyme (ACE) inhibitor, which exerts a well-established antiremodeling action in several organs and various pathologies (Pfeffer et al., 1992; Pechánová et al., 1997; Simko et al., 2010, 2014; Repová-Bednárová et al., 2013) including lactacystin-induced LV remodeling (Simko et al., 2017).

## Materials and methods

### Animals and treatment

The experiments were conducted in conformance with the Guide for the Care and Use of Laboratory Animals published by the US National Institutes of Health (NIH Publication No. 85-23, revised 1996). The study protocol was approved by the ethics committee of the Institute of Pathological Physiology, Faculty of Medicine, at Comenius University in Bratislava, Slovakia.

Thirty-two adult (12-week-old) weight-matched male Wistar rats (obtained from the Department of Toxicology and Laboratory Animals Breeding, Slovak Academy of Sciences, Dobra Voda, Slovakia) were randomly divided into four groups (*n* = 8 per group) and treated for 6 weeks as follows: control (C; untreated), lactacystin (Lac; 5 μg/kg/day), lactacystin plus captopril (Lac + Cap; 5 μg/kg/day lactacystin + 100 mg/kg/day captopril), and lactacystin plus melatonin (Lac + Mel; 5 μg/kg/day lactacystin + 10 mg/kg/day melatonin). Lactacystin, captopril, and melatonin were dissolved in drinking water and their concentrations were adjusted to daily water consumption to ensure the correct dosage. The melatonin solutions were offered in non-transparent bottles to protect them from light.

Lactacystin and melatonin were purchased from Sigma-Aldrich Chemie, Munich, Germany, and captopril from Egis Pharmaceuticals, Budapest, Hungary.

The rats were housed in individual cages, maintained under standard laboratory conditions (12:12-h light-dark cycle at 22–24°C temperature and 45%–65% humidity), and fed a regular pelleted diet *ad libitum*. Systolic blood pressure (SBP) was measured once a week in each animal by non-invasive tail-cuff plethysmography (Hugo-Sachs Elektronik, Freiburg, Germany). After 6 weeks of treatment, the rats were euthanized by isoflurane inhalation. Two halves of the left kidney were harvested for subsequent analyses: one half was fixed in 4% formaldehyde for histopathological analysis. The other was snap-frozen at −80°C for determination of hydroxyproline concentration.

### Kidney histopathology

The kidney samples fixed in 4% formaldehyde were embedded in paraffin and cut into 5 μm-thick sections. Then, two sets of sections per sample were deparaffinized, rehydrated, and stained, one with hematoxylin-eosin (H-E) for glomerular morphometry, and the other with picrosirius red (PSR) for quantitative analysis of kidney fibrosis. Photomicrographs were taken using a NIKON Eclipse Ti C2+ microscope (NIKON, Tokyo, Japan) with transmitted or polarized light and subsequently analyzed with NIKON NIS-Elements Analysis software (NIKON, Tokyo, Japan) and ImageJ version 1.52p for Windows (National Institutes of Health, Bethesda, MD, United States).

The H-E-stained sections were analyzed using transmitted light microscopy at ×10 magnification and NIKON NIS-Elements Analysis software to assess glomerular morphometry, as previously described (Pechanova et al., 2006; Hrenák et al., 2013; Stanko et al., 2020). Glomerular density was determined by counting preserved glomeruli in a 1 mm^2^ digital frame put over the kidney cortex at 10 microscopic fields per section; i.e., 80 digital frames were investigated per group. The glomerular tuft area was determined by measuring perpendicular maximum and minimum diameters (d_max_ and d_min_, respectively) of 10 random glomerular tufts per section used for subsequent tuft area calculation as follows: glomerular tuft area = π(d_max_/2) (d_min_/2); i.e., 80 glomerular tuft areas were calculated per group.

The PSR-stained sections were analyzed with polarized light microscopy, set at ×100 magnification, and ImageJ to allow for a quantitative assessment of kidney fibrosis, as previously described (Seccia et al., 2008; Stanko et al., 2020). Due to the birefringence shift by PSR, the thick type I collagen (Col I) was shown in red-orange shades, and the thin type III collagen (Col III) was visualized in green-yellow shades. Thus, the Col I and Col III volumes were determined as the percentage of red-orange and green-yellow shaded areas in a particular region of interest (ROI) by setting the appropriate “hue” thresholds of the color spectrum in ImageJ. To assess glomerular fibrosis, 40 ROIs per section of 50 × 50 μm were placed in intraglomerular space; i.e., 320 intraglomerular ROIs were investigated per group. To assess tubulointerstitial fibrosis, 40 ROIs per section of 192 × 72 μm were placed at the interstitial cortex without glomeruli or vessels; i.e., 320 tubulointerstitial ROIs were investigated per group. To evaluate the amount of vascular/perivascular fibrosis, five ROIs per section were examined, tight-cropping a cross-section captured artery with a diameter between 50 and 100 μm; i.e., 40 vascular/perivascular ROIs were investigated per group.

The histopathological analysis was performed by an experienced investigator blinded to the group identity.

### Determination of kidney hydroxyproline concentration

The kidney samples, snap-frozen and stored at −80°C, were dried at 100°C for 24 h and then hydrolyzed using a solution of 6 mol/L HCl. The hydroxyproline concentration was determined spectrophotometrically at 550 nm (Reddy and Enwemeka, 1996; Hrenák et al., 2013).

### Statistical analysis

The results are presented as the mean ± SEM. The one-way, two-tailed analysis of variance (ANOVA), followed by a Holm-Sidak multiple comparisons test, was used for statistical analysis. *p* values below 0.05 were considered statistically significant. The statistical analysis was conducted using GraphPad Prism 8 for Windows (GraphPad Software, La Jolla, CA, United States).

## Results

### Systolic blood pressure

**FIGURE 1:**
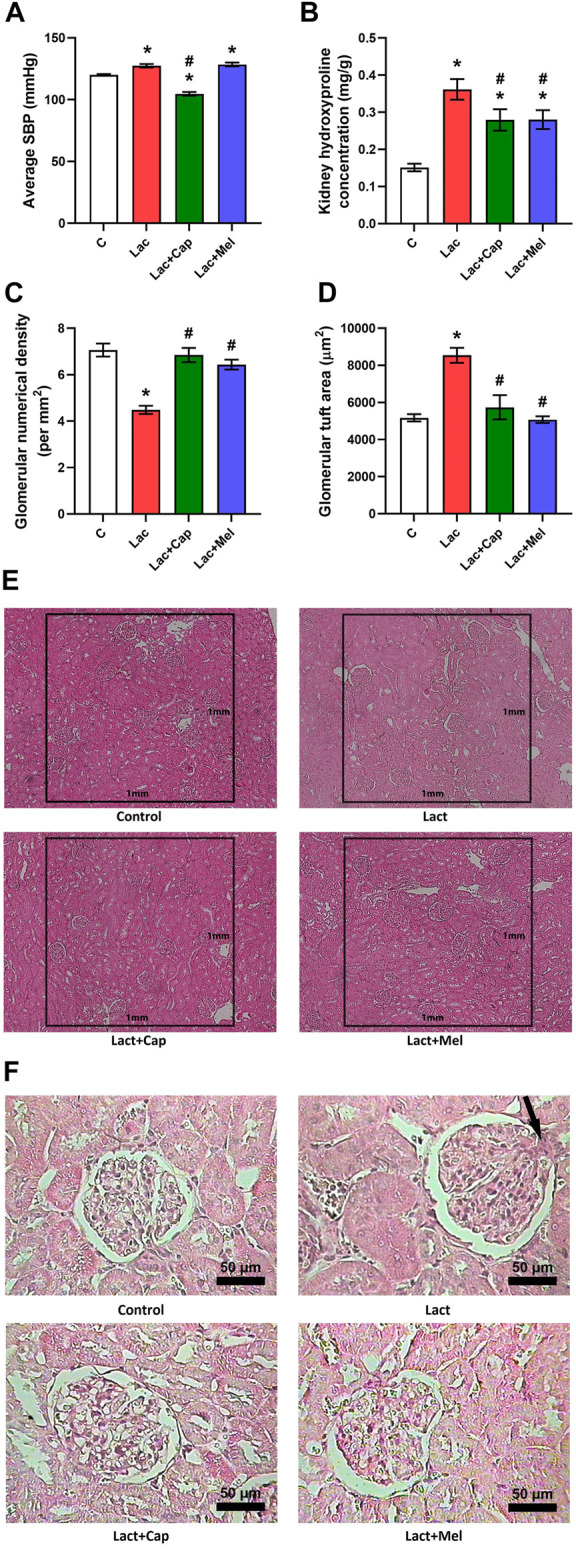
Effect of captopril (Lac + Cap) and melatonin (Lac + Mel) on average systolic blood pressure (SBP) **(A)**, renal hydroxyproline concentration **(B)**, and kidney morphology: glomerular numerical density per 1 mm^2^ **(C)** and glomerular tuft area **(D)** of lactacystin-treated (Lac) rats; glomerular numerical density **(E)** and detailed images of the glomerular content **(F)** in lactacystin-treated rats: H-E-stained sections at ×10 **(E)** and ×100 **(F)** magnification using transmitted light microscopy; mesangial cell proliferation with extracellular matrix expansion (arrow). C, controls; *p < 0.05 vs. C; ^#^ *p* < 0.05 vs. Lac.

The SBP averaged over 6 weeks of treatment was 120.07 ± 0.58 mmHg in controls, and lactacystin raised the value by 6% (*p* < 0.05). In the lactacystin group, captopril lowered the average SBP by 18% (*p* < 0.05), while melatonin had no effect on average SBP (Figure 1A).

### Glomerular morphometry

The glomerular numerical density was 7.06 ± 0.28 per mm^2^ in controls; lactacystin decreased this value by 37% (*p* < 0.05). In the lactacystin group, both captopril and melatonin augmented the glomerular numerical density by 53% and 44% (*p* < 0.05 for both measures), respectively (Figures 1C,E).

The glomerular tuft area was 5.173 ± 198 μm^2^ in controls, and lactacystin increased this value by 65% (*p* < 0.05). In the lactacystin group, both captopril and melatonin reduced the glomerular tuft area by 33% and 41% (*p* < 0.05 for both measures), respectively (Figures 1D,F).

### Kidney hydroxyproline concentration

The kidney hydroxyproline concentration was 0.15 ± 0.05 mg/g in controls, and lactacystin raised (*p* < 0.05) it by 139%. In the lactacystin group, both captopril and melatonin lowered (*p* < 0.05) the kidney hydroxyproline concentration by 23% (Figure 1B).

### Quantitative analysis of kidney fibrosis

**FIGURE 2:**
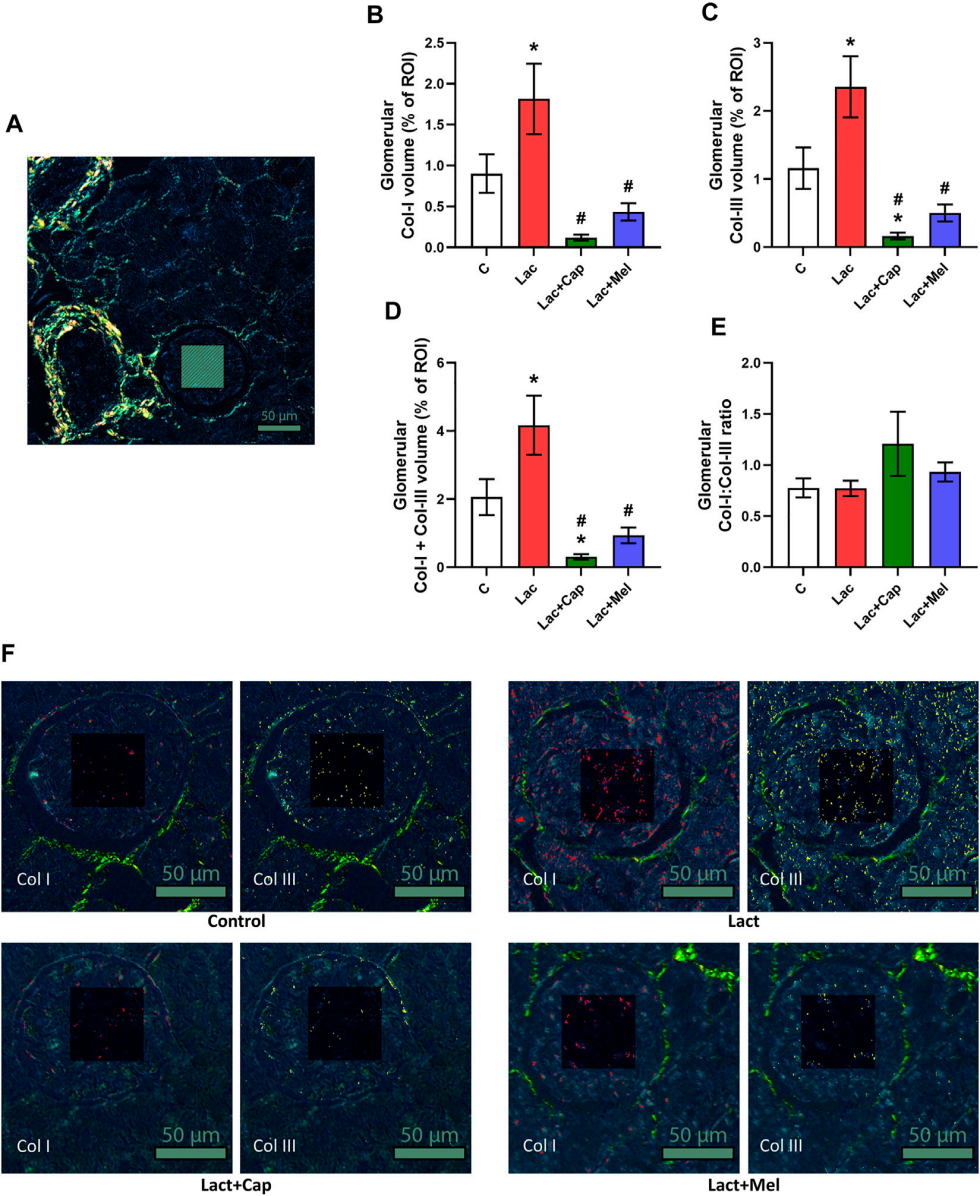
Effect of captopril (Lac + Cap) and melatonin (Lac + Mel) on glomerular fibrosis of lactacystin-treated (Lac) rats. PSR-stained section at ×100 magnification using polarized light microscopy **(A)**, the volume of collagen I (Col I) **(B)**, collagen III (Col III) **(C)**, the sum of collagen I + III **(D)**, the Col I/Col III ratio **(E)** and PSR-stained sections at ×200 magnification using polarized microscopy showing collagen I in red and collagen III in yellow **(F)**. C, controls; ROI, region of interest depicted as the shaded rectangle. Intraglomerular ROI dimensions: 50 μm × 50 μm. Scale bar: 50 μm. *p < 0.05 vs. C; ^#^ *p* < 0.05 vs. Lac.

For intraglomerular ROIs, the volume of Col I and Col III in controls was 0.90 ± 0.23 and 1.16 ± 0.30%, respectively. Lactacystin increased the proportion of both Col I and Col III by 101% and 103% (*p* < 0.05 for both measures), respectively. In the lactacystin group, captopril lowered the proportion of both Col I and Col III by 93% (*p* < 0.05 for both measures). Similarly, melatonin reduced the proportion of both Col I and Col III by 76% and 79% (*p* < 0.05 for both measures), respectively. The sum of Col I and Col III volume in intraglomerular ROIs was 2.06 ± 0.53% in controls, and lactacystin increased this value by 102% (*p* < 0.05). In the lactacystin group, both captopril and melatonin decreased the sum of Col I and Col III volume by 93% and 78% (*p* < 0.05 for both measures), respectively. The ratio of Col I to Col III (Col I/Col III) was 0.78 ± 0.09 in controls, and neither lactacystin, captopril, nor melatonin had any effect on the ratio (Figure 2).

**FIGURE 3:**
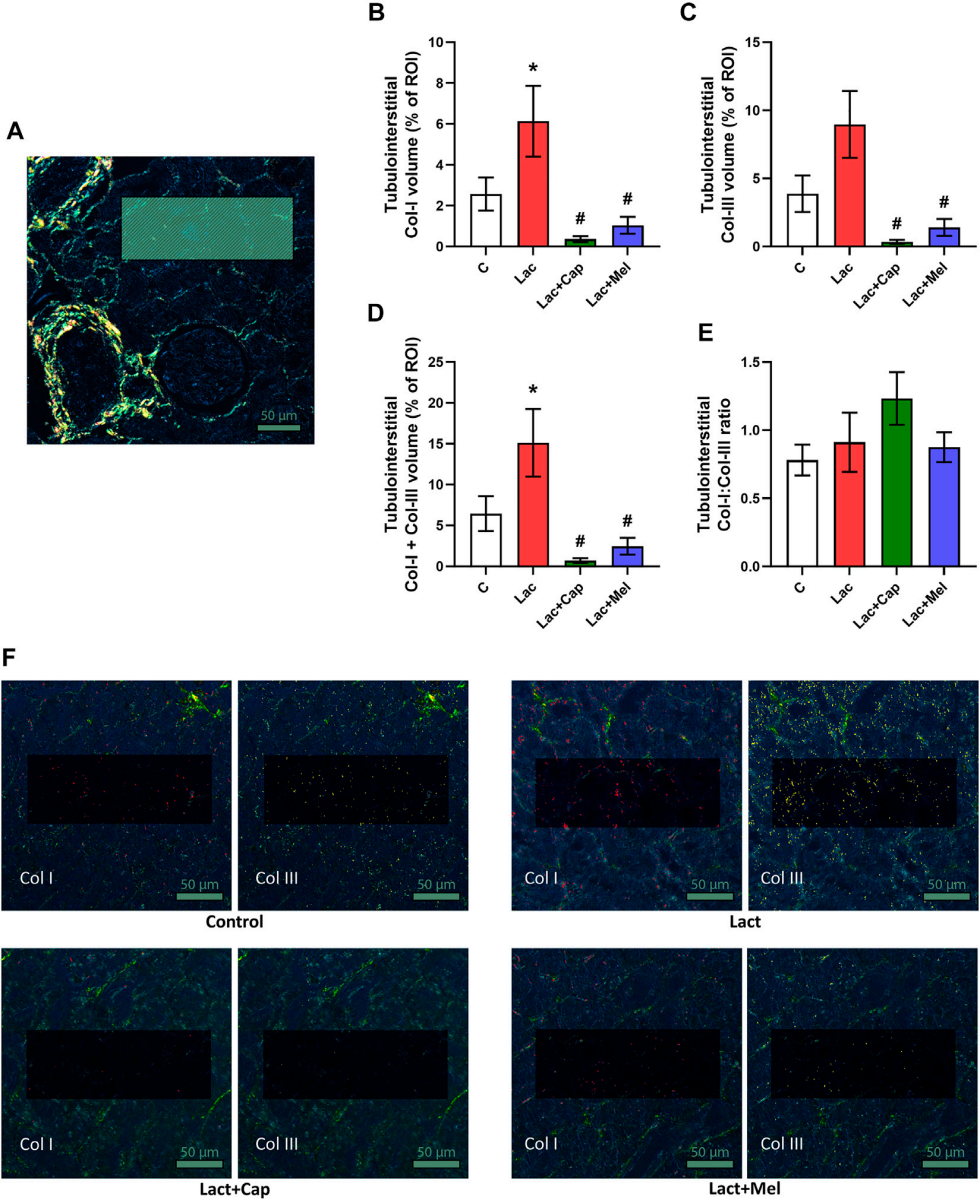
Effect of captopril (Lac + Cap) and melatonin (Lac + Mel) on tubulointerstitial fibrosis of lactacystin-treated (Lac) rats. PSR-stained section at ×100 magnification using polarized light microscopy **(A)**, the volume of collagen I (Col I) **(B)**, collagen III (Col III) **(C)**, the sum of collagen I + III **(D)**, the Col I/Col III ratio **(E)** and PSR-stained sections at ×100 magnification using polarized microscopy showing collagen I in red and collagen III in yellow **(F)**. C, controls; ROI, region of interest depicted as the shaded rectangle. Tubulointerstitial ROI dimensions: 72 μm × 192 μm. Scale bar: 50 μm. *p < 0.05 vs. C; ^#^ *p* < 0.05 vs. Lac.

For tubulointerstitial ROIs, the volume of Col I and Col III in controls were 2.57 ± 0.81 and 3.88 ± 1.34%, respectively. Lactacystin increased the proportion of Col I by 139% (*p* < 0.05) and enhanced the proportion of Col III by 131% (ns). In the lactacystin group, captopril reduced the proportion of both Col I and Col III by 94% and 96% (*p* < 0.05 for both measures), respectively. Similarly, melatonin decreased the proportion of both Col I and Col III by 83% and 84% (*p* < 0.05 for both measures), respectively. The sum of Col I and Col III volume in tubulointerstitial ROIs was 6.45 ± 2.13% in controls, and lactacystin increased this value by 134% (*p* < 0.05). In the lactacystin group, both captopril and melatonin reduced the sum of Col I and Col III volume by 95% and 84% (*p* < 0.05 for both measures), respectively. Col I/Col III was 0.78 ± 0.11 in controls, and lactacystin, captopril, or melatonin had no effect on the ratio (Figure 3).

**FIGURE 4:**
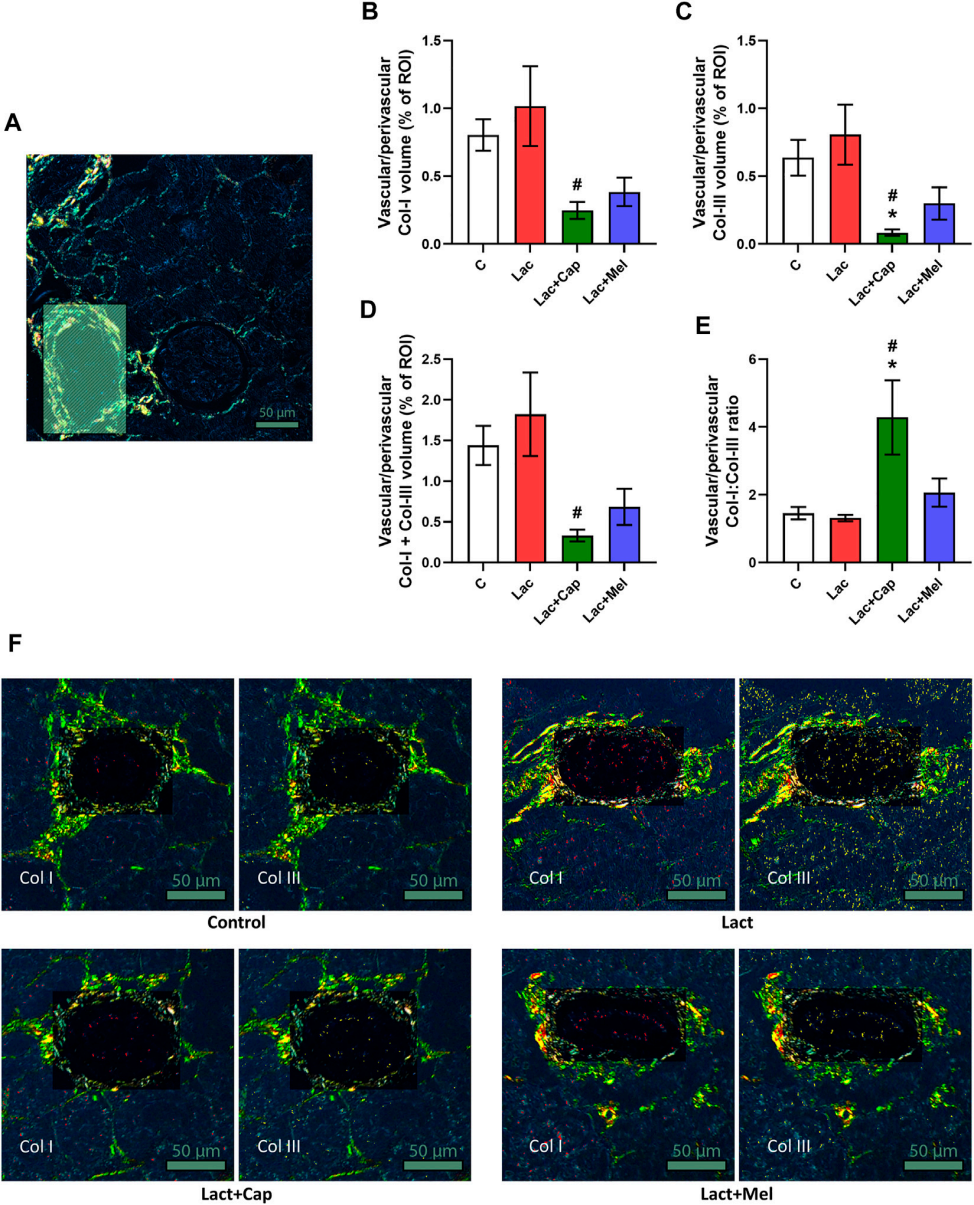
Effect of captopril (Lac + Cap) and melatonin (Lac + Mel) on vascular/perivascular fibrosis of lactacystin-treated (Lac) rats. PSR-stained section at ×100 magnification using polarized light microscopy **(A)**, the volume of collagen I (Col I) **(B)**, collagen III (Col III) **(C)**, the sum of collagen I + III **(D)**, the Col I/Col III ratio **(E)** and PSR-stained sections at ×100 magnification using polarized microscopy showing collagen I in red and collagen III in yellow **(F)**. C, controls; ROI, region of interest depicted as the shaded rectangle. Vascular/perivascular ROI dimensions: from 50 × 100 μm to 200 × 300 μm. Scale bar: 50 μm. *p < 0.05 vs. C; ^#^ *p* < 0.05 vs. Lac.

In vascular/perivascular ROIs, Col I and Col III’s volume in controls was 0.80 ± 0.12 and 0.64 ± 0.13%, respectively. Lactacystin had no significant effect on the volume of Col I and Col III. In the lactacystin group, captopril reduced the proportion of both Col I and Col III by 76% and 90% (*p* < 0.05 for both measures), respectively, while melatonin had no effect. The sum of Col I and Col III volume in vascular/perivascular ROIs was 1.44 ± 0.24% in controls, and lactacystin did not significantly change this parameter. In the lactacystin group, captopril decreased the sum of Col I and Col III volume by 82% (*p* < 0.05), and melatonin had no effect. Col I/Col III was 1.45 ± 0.18 in controls, and lactacystin had no significant effect on the ratio. In the lactacystin group, captopril increased Col I/Col III ratio by 226% (*p* < 0.05), and melatonin had no effect (Figure 4).

## Discussion

Six weeks of lactacystin administration raised the average SBP. In kidneys, lactacystin reduced glomerular density, increased the glomerular tuft area, and enhanced the hydroxyproline concentration. Lactacystin likewise elevated the intraglomerular proportion and the sum of Col I and Col III, the tubulointerstitial proportion of Col I and the sum of Col I and Col III without an effect on vascular/perivascular collagen. Six weeks of captopril treatment reduced SBP, while melatonin had no effect. Both melatonin and captopril raised glomerular density, reduced the glomerular tuft area, and decreased hydroxyproline concentration in the kidneys. Both drugs reduced the proportion and sum of intraglomerular and tubulointerstitial Col I and Col III.

Since the UPS controls the protein turnover of both regulatory and structural proteins, it becomes an attractive target for pharmacological interventions. Lactacystin represents the classical, first discovered proteasome inhibitor, reported in 1991 (Ōmura and Crump, 2019). Among other effects, lactacystin is considered to be an inhibitor of nuclear factor kappa B (NF-κB) transcription factor. Since NF-κB is assumed to be a checkpoint for hypertrophic growth mediated by humoral factors, inhibitors of NF-κB, such as lactacystin, are considered a possible way of protection. The net effect of lactacystin is challenging to estimate for two reasons: first, NF-κB is not only an essential factor for proliferation (Bellas et al., 1995); it may also interfere with nitric oxide synthesis, having the opposite effect (Vrankova et al., 2010; Simko et al., 2017). Moreover, lactacystin not only specifically blocks the degradation of the NF-κB inhibitor IκBα but can also modify other proteins involved in signaling processes (Vrankova et al., 2010).

In line with this, surprisingly, lactacystin did not reduce but increased blood pressure and fibrotic rebuilding in the left ventricle (Simko et al., 2017). Thus, lactacystin administration-induced BP elevation was recently characterized as a novel model of experimental hypertension (Vrankova et al., 2010; Simko et al., 2017; Sharma et al., 2020). Proteasome inhibition may cause hypertension either because of an increased endogenous protein inhibitor of neuronal nitric oxide synthase, leading to decreased NO bioavailability in the paraventricular nucleus (Sharma et al., 2020), or from tyrosine hydroxylase upregulation and activation in the hypothalamus and brainstem (Congo Carbajosa et al., 2015), both resulting in increased sympathetic outflow. Bearing in mind the remodeling of the heart (Simko et al., 2017) and aorta (Vrankova et al., 2010) in this model, it seems to be of importance to disclose whether lactacystin could act in a similar pro-proliferative way in hypertensive kidneys.

In this experiment, chronic lactacystin treatment was associated with the loss of glomeruli, indicated by decreased glomerular density and simultaneous glomerular hypertrophy, reflected in increased glomerular tuft area. Some authors consider the reduction of nephron density to be a risk factor for hypertension and CKD progression (Kanzaki et al., 2020). Furthermore, lactacystin treatment was associated with enhanced hydroxyproline concentration and site-specific fibrotic rebuilding of renal tissue. Published data suggest that chronic hypertension leads to the accumulation and dysregulation of extracellular matrix in the kidneys, resulting in renal fibrosis (Schlondorff, 2008). The fibrotic changes in the kidneys are common 1) in the glomerulus—glomerulosclerosis; 2) in the tubulointerstitium—interstitial fibrosis; and 3) in the vessels—arteriosclerosis and perivascular fibrosis (Bülow and Boor, 2019). The most abundant collagens expressed in the kidneys are types I and III. Col I forms long, thick, and stiff fibrils, decreasing tissue compliance (Sopakayang et al., 2012). In the early stages of renal fibrosis, Col I deposits in the glomerulus, tubulointerstitial space, and arterial wall (Alexakis et al., 2006). Col III is mainly found in softer tissues and is more distensible than Col I (Silver et al., 2001). In renal fibrosis, the expression of Col III increases in both interstitium and glomeruli (Alexakis et al., 2006). In the present study, an excessive deposition of Col I and Col III was observed in the glomerulus and tubulointerstitial space in lactacystin-treated hypertensive rats.

ACE inhibitor captopril reduces fibrosis associated with target organ damage. Indeed, captopril decreased the concentration of soluble collagen in the LV of rats with combined continuous light and L-NAME-induced hypertension (Simko et al., 2010), attenuated LV collagen deposition in SHR (Zhao et al., 2015), and reduced LV fibrosis in mice with transverse aortic constriction (Zhang et al., 2019) and Sprague-Dawley rats with L-NAME-induced hypertension (Sonoda et al., 2017). Similarly, in kidneys captopril reduced interstitial renal fibrosis in neonatal dogs with partial urethral obstruction (PUO) (Shirazi et al., 2007), ameliorated fibrosis in rats with PUO (Shirazi et al., 2014), and downregulated interstitial fibrosis in rats with unilateral ureteral obstruction (Hosseinian et al., 2019). In line with these data, our results show reduced intraglomerular, tubulointerstitial, and perivascular collagen accumulation and renal hydroxyproline concentration after 6 weeks of captopril administration in lactacystin-treated rats. In this experiment, captopril reduced the proportion and sum of intraglomerular and tubulointerstitial Col I and Col III, lowered the sum of vascular/perivascular Col I and Col III and increased the vascular/perivascular ratio of Col I to Col III, while reducing the kidney hydroxyproline concentration.

Melatonin exerts a vast number of pleiotropic protective effects on various tissues, including fibrosis amelioration. The antifibrotic effects of melatonin have been observed in the LV of lactacystin-treated rats (Simko et al., 2017), of SHRs (Simko et al., 2009), and in the LV and aorta of 24-h continuous light-exposed rats (Repová-Bednárová et al., 2013; Simko et al., 2014). In the kidneys, melatonin reduced renal fibrosis in mice with unilateral ureteral obstruction (Li et al., 2020), adenine-induced CKD mice (Yoon et al., 2020), diabetic mice (Li et al., 2019; Fan et al., 2020), prenatally diclofenac sodium injected rats (Khoshvakhti et al., 2015), and in human renal proximal tubule epithelial cells on a high glucose diet (Han et al., 2020). Accordingly, in this study, 6 weeks of melatonin treatment prevented intraglomerular and tubulointerstitial fibrosis development, alongside reduced renal hydroxyproline concentration in rats administered lactacystin. Although melatonin failed to reduce SBP in this experiment, its antifibrotic effects were likely a result of melatonin’s direct, local actions, such as obvious antioxidant and radical scavenging actions (Simko et al., 2009; Reiter et al., 2018), modulation of the sympathetic and renin-angiotensin system (Simko and Paulis, 2013), and increasing NO bioavailability and antiproliferative action (Paulis and Simko, 2007; Simko and Paulis, 2007, 2013; Simko and Pechanova, 2009; Domínguez-Rodríguez et al., 2021).

The lactacystin administration induced fibrotic rebuilding of the kidneys. Both captopril and melatonin reduced the proportion and sum of intraglomerular and tubulointerstitial Col I and Col III and hydroxyproline concentration in the kidneys. Although captopril more prominently reduced Col I and Col III accumulation in the vascular/perivascular area compared to melatonin, the Col I/Col III ratio rose. Considering the stiffness of Col I, the findings suggest that captopril might cause renal capillaries to become more rigid, altering renal perfusion. On the contrary, melatonin did not significantly change the renal vascular/perivascular fibrosis while maintaining a normal Col I/Col III ratio. Since it concurrently reduced glomerular fibrosis, melatonin may play a role in maintaining adequate glomerular perfusion and filtration. Thus, melatonin may be comparable or superior to ACE inhibition because of its distinct effect on renal collagen composition, potentially contributing to improved renal perfusion and filtration rate.

## Conclusion

In a model of lactacystin-induced hypertension and organ damage, both melatonin and captopril raised glomerular density, reduced the glomerular tuft area, and lowered the kidney hydroxyproline concentration. Both drugs reduced the proportion and sum of intraglomerular and tubulointerstitial Col I and Col III. As melatonin failed to reduce SBP, its antifibrotic effects were supposedly delivered by melatonin’s direct, local pleiotropic effects.

Considering the fibrotic remodeling of the left ventricle observed in previous works and the site-specific fibrotic rebuilding of the kidneys observed in this study, it seems reasonable to suggest that chronic treatment with lactacystin may be a perspective model of cardiorenal damage. Furthermore, we showed that melatonin, similar to captopril, is a promising means of protection against hypertensive kidney damage.

## Limitations

It would be of interest to correlate the renal histopathologic findings with immunohistochemical analysis of growth factors, angiogenic factors, and markers of collagen turnover in the kidneys. Indeed, according to the literature, there is a correlation between the histopathological evidence of renal fibrosis and profibrotic renal tissue markers, such as transforming growth factor-β1 (TGF-β1), connective tissue growth factor (CTGF), and vascular endothelial growth factor A (VEGF-A) (Lopes et al., 2019). Moreover, the inhibition of matrix metalloproteinase (MMP)-9 (Wang et al., 2019) and decreased expression of MMP-1 (Nazneen et al., 2002) resulted in decreased histological evidence of renal fibrosis. However, these analyses were beyond the scope and possibilities of the present histopathological study.

## Data Availability

The raw data supporting the conclusion of this article will be made available by the authors, without undue reservation.
